# Smart Speakers for Health and Well-Being of Older Adults: A Mixed-Methods Review

**DOI:** 10.3390/healthcare13212772

**Published:** 2025-10-31

**Authors:** Michael Joseph Dino, Carla Leinbach, Gerald Dino, Ladda Thiamwong, Chloe Margalaux Villafuerte, Mona Shattell, Justin Pimentel, Maybelle Anne Zamora, Anbel Bautista, John Paul Vitug, Joyline Chepkorir, Nerceilyn Marave

**Affiliations:** 1 College of Nursing, University of Central Florida, 6825 Lake Nona Blvd, Orlando, FL 32827, USAmona.shattell@ucf.edu (M.S.); 2 Research Development and Innovation Center, Our Lady of Fatima University, 120 McArthur Highway, Marulas, Valenzuela City 1440, Philippinesmlzamora@fatima.edu.ph (M.A.Z.);; 3 The Libraries, De La Salle University, 2401 Taft Avenue, Malate, Manila 0922, Philippines; gerald.dino@dlsu.edu.ph; 4 Center for Cardiovascular and Chronic Care, School of Nursing, Johns Hopkins University, Baltimore, MD 21205, USA

**Keywords:** smart speakers, older adults, aging in place, mixed-methods review

## Abstract

**Background:** Rapid population aging poses significant challenges to health and wellness systems, necessitating innovative technological interventions. Smart home technologies, particularly voice-activated intelligent assistants (smart speakers), represent a promising avenue for supporting aging populations. **Objectives:** This study critically examines the empirical literature on smart speakers’ influence on older adults’ health and well-being, mapping the characteristics of existing studies, assessing the current state of this domain, and providing a comprehensive overview. **Methods:** A mixed-methods systematic review was conducted in accordance with published guidelines. Bibliometric data, article purposes and outcomes, keyword network analysis, and mixed-methods findings from articles retrieved from five major databases were managed through the Covidence and VosViewer applications. **Results:** The majority of studies were conducted in the American region. Bibliometric analysis revealed five predominant thematic clusters: health management, psychological support, social connectedness, technology adoption, and usability. Findings demonstrated multifaceted benefits across several domains. Older adults reported improvements in daily living activities, enhanced emotional well-being, strengthened social connections, and overall health benefits. Qualitative evidence particularly emphasized the advantages of medication adherence, routine maintenance, and facilitated social support. However, mixed-method synthesis revealed significant barriers to adoption and sustained use, including privacy concerns, technical difficulties, cost constraints, and limited digital literacy among older users. **Conclusions:** The integration of smart speakers into the homes of older adults offers considerable potential to enhance technological wellness and promote successful aging in place, underscoring the need for structured integration of smart speaker technology and human-centered designs within geriatric care systems.

## 1. Introduction

The global population of older adults is projected to experience significant growth over the next three decades. By 2050, studies indicate that approximately 1.5 billion individuals aged 65 and older will be residing worldwide [1,2]. This rapid aging presents significant challenges, necessitating innovative approaches to promote health and wellness in this demographic. To address these challenges, supporting older adults in aging in place within their homes and communities has become a key policy priority [3]. This approach is aligned with the currently accepted framework [4], which emphasizes that well-being is defined by the “beings” and “doings” of daily life [5]. Quality of life, in this context, has been assessed based on the ability to achieve these valued functions [6]. By integrating these principles into public health strategies specifically designed for older adults, prevalent issues of isolation and functional decline often experienced by this population may be mitigated [7].

The emergence of advanced technologies, particularly smart home systems, has played a vital role in supporting aging in place. One key example is the smart speaker, which has become a valuable tool in the care of older adults [8,9]. This voice-activated device performs various tasks with simple spoken commands, making it particularly useful for older adults seeking to simplify their daily routines. They assist with everyday activities such as setting medication reminders, checking the weather, playing familiar music, or calling family members, without requiring users to navigate complex interfaces or small buttons. Popular models, including Amazon Echo, Apple HomePod, and Google Home, provide hands-free convenience that is particularly beneficial for individuals with mobility limitations or visual impairments.

While these functions are widely recognized, smart speaker applications differ significantly across regions and cultural contexts. For instance, Eastern Asian users often integrate smart speakers into social, educational, and family-centered activities [10]. In contrast, in developing regions, usage is shaped by factors such as language localization, affordability, and access to digital infrastructure, with devices often serving educational or informational purposes [11]. These cultural and contextual variations highlight the need to understand how smart speakers function not only as technological tools but also as socially embedded devices.

Previous studies have additionally demonstrated that these devices can be effectively integrated into health and wellness initiatives for older adults. These devices feature enhanced programs designed to increase physical activity [12] and support self-management and care practices [7,10]. Research indicates that older adults often form emotional attachments to smart speakers, thereby further integrating these devices into their daily routines and enhancing their overall well-being [8,13]. Ultimately, these devices offered an accessible entry point into helpful technology for older adults seeking to stay connected and maintain their independence, eliminating the learning curve associated with smartphones or computers [14].

Despite these promising developments, significant gaps remain. There is limited understanding of the distinct and collective effectiveness of smart speakers in empirical contexts, especially across qualitative, quantitative, and mixed-methods studies. In response, the present study critically examines published empirical research on the impact of smart speakers on the health and well-being of older adults. Specifically, it seeks to map the characteristics of existing studies, assess the progression and current state of this domain, and conduct a mixed-methods analysis to provide a comprehensive overview.

The present study is guided by the Technology Acceptance Model (TAM) by Davis (1989) [15], which provides a robust framework for understanding how individuals come to adopt and use new technologies. TAM conveys that two primary factors influence technology acceptance: perceived usefulness (i.e., the degree to which a person believes that using the technology will enhance their performance or daily life) and perceived ease of use or the degree to which a person believes that using the technology will be free of effort. In the context of older adults, these constructs are particularly relevant, as adoption decisions are influenced not only by the functionality of smart speakers but also by the simplicity of interaction and the perceived benefits in daily living, health management, and social connectivity.

## 2. Materials and Methods

This project employed the Segregated Synthesis Mixed-Methods review process [16]. The sequential steps included developing the mixed-methods research question, conducting a comprehensive search for relevant papers, critically appraising the identified studies, extracting data, performing qualitative or quantitative meta-synthesis, and synthesizing the mixed-methods findings. The authors have ensured adherence to the Preferred Reporting Items for Systematic Reviews and Meta-Analysis (PRISMA) guidelines [17] throughout the process.

### 2.1. Stage 1: Developing the Mixed-Methods Research Question

Three key elements were considered in formulating the research questions, namely: (1) older adults (the target population), (2) the use of smart speakers (the intervention), and (3) health-related outcomes. The following research questions guided this mixed-methods review:

1. What characterizes the existing empirical articles on interventions utilizing smart speakers among older adult populations? (*article bibliometrics*)
2. What health-related outcomes have been associated with the use of smart speakers by older adults? (*quantitative synthesis*)
3. What are the reported experiences, perceptions, barriers, and facilitators by the older adults regarding the use of smart speakers? (*qualitative synthesis*)
4. To what extent do qualitative and quantitative findings align in terms of the effectiveness and acceptability of smart speakers in enhancing health outcomes for older adults? (*mixed-method synthesis*)

### 2.2. Stage 2: Search for Papers

A comprehensive literature search was conducted across Scopus, Web of Science, PubMed, ProQuest, and IEEE databases using the keywords “older adult”, “smart speaker”, “smart audio”, “health”, “physical”, “mental”, and “well-being” (see Table 1). The literature search focused exclusively on peer-reviewed empirical studies published in academic journals. Gray literature sources, such as theses, reports, and policy briefs, were excluded to maintain methodological consistency and ensure that all included studies could be appraised using standardized criteria (MMAT).

The following inclusion criteria have been used in selecting related articles:

#### 2.2.1. Population

The target population for this review consisted of individuals aged 65 and older, commonly referred to as older adults. Various age-related physiological changes and an increased risk and prevalence of chronic diseases characterize this age group. Older adults frequently confront social challenges, including social isolation and reduced mobility, which necessitate tailored interventions [18]. The selection of older adults as the target population for this review was based on their distinct demographic and health-related needs, making them a critical population for initiatives leveraging smart speaker technology [7]. Therefore, this review focused specifically on articles that addressed the needs of older adults as the primary population of concern.

#### 2.2.2. Interventions

This review primarily focused on articles exploring the implementation of smart speaker interventions. This technology offers functionalities that facilitate easier interaction with the digital world, making it more appealing to older adults who may struggle with complex technological interfaces [10]. Articles that examined interventional strategies using smart speakers tailored to older adults were included in this review.

#### 2.2.3. Context and Study Design

This review demonstrated broad temporal and geographic inclusivity, with a preference for English-language articles. Its scope encompasses interventions delivered via smart speakers in healthcare and community-based settings, including, but not limited to, primary care environments such as clinics, community hospitals, nursing homes, and assisted living facilities. Eligible for review were all empirical studies that examine outcomes associated with the implementation of smart speakers, employing quantitative, qualitative, or mixed-methods research designs. Quantitative studies have included randomized and non-randomized controlled trials, as well as pre-post studies with and without control groups. Qualitative studies exploring participants’ perspectives or experiences through diverse qualitative methodologies, such as phenomenology or case studies, have been included. Theoretical studies, protocol papers, reviews, and quality improvement articles have been excluded.

#### 2.2.4. Outcomes

This review aimed to examine the health-related outcomes associated with interventions utilizing smart speaker technology. By comprehensively analyzing a range of health domains, this review aimed to elucidate which specific domain was prominently prioritized based on current evidence. Through this nuanced exploration, a deeper understanding of the potential impact of smart speakers on various health outcomes has been discovered.

**Figure 1:**
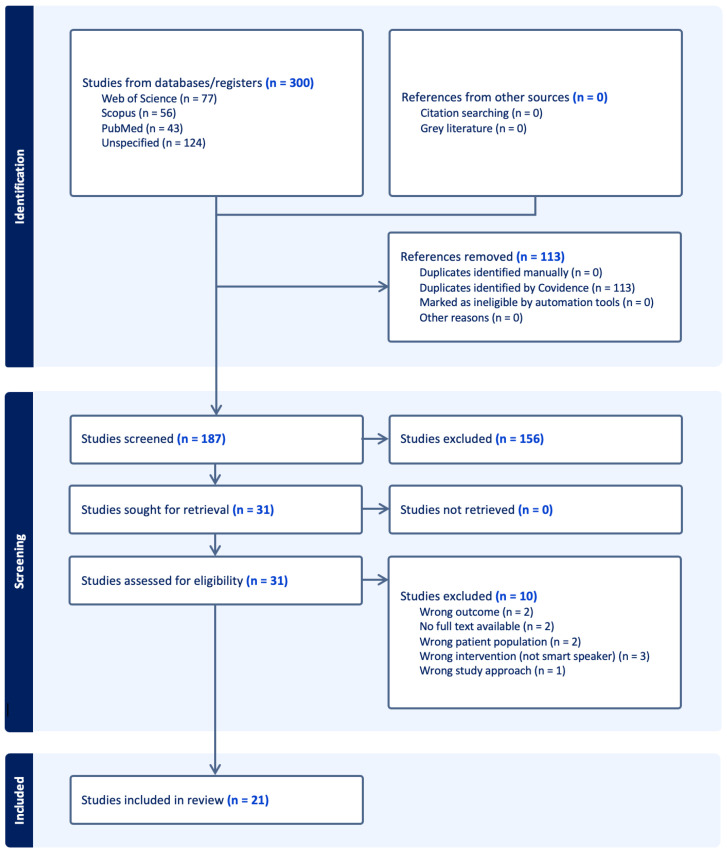
PRISMA Diagram.

Articles specifically pertaining to adolescents or other population groups that utilized robotics and chatbot technology were systematically excluded from this study. A total of 300 retrieved articles were imported into Covidence and VOSviewer (version 1.6.20) to facilitate screening, data extraction, and subsequent analysis. To ensure reliability and mitigate potential bias, at least two reviewers screened each article at every stage of the review process. The principal investigator acted as a conflict resolver, facilitated the discussion, and rendered the final decision based on group consensus. The review process started with the uploading of articles, followed by the removal of one hundred and three (n = 103) duplicates. One hundred and eighty-seven (n = 187) articles underwent initial screening of titles and abstracts, while one hundred and fifty-six irrelevant articles were removed. Thirty-one (n = 31) studies were eligible for a comprehensive review of full texts. Ten (n = 10) studies were excluded for various reasons, leaving 21 articles eligible for critical appraisal and data extraction (see Figure 1).

The PRISMA diagram (Figure 1) illustrates the article screening process using Covidence. In total, 300 studies were included, with 113 duplicates automatically removed by the application. Among the 187 articles screened, 156 were excluded at the title or abstract level because they did not meet the inclusion criteria discussed in stage two. Thirty-one articles underwent a full-text review, of which 11 were excluded due to incorrect outcomes, patient populations, interventions, or study approaches, or because full-text papers were unavailable. Ultimately, 20 studies met all eligibility criteria and were included in the mixed-methods review.

### 2.3. Stage 3: Critical Appraisal

The authors utilized the Mixed-Methods Assessment Tool (MMAT) to evaluate the quality and publication bias of the articles (i.e., small or non-representative samples, reliance on cross-sectional designs, and limited reporting of qualitative rigor). Data were synthesized using the standards published [16]. For qualitative studies, criteria assessed included: (1) the appropriateness of the qualitative approach, (2) adequacy of data collection methods, (3) derivation of findings from the data, (4) substantiation of interpretations by the data, and (5) coherence between data sources, collection, analysis, and interpretation. For quantitative studies, the criteria examined varied by design type but generally included (1) representativeness of the sample, (2) appropriateness of measurements, (3) completeness of outcome data, (4) control for confounding factors, and (5) appropriateness of statistical analyses. Finally, for mixed-methods studies, additional criteria assessed (1) rationale for using mixed methods, (2) integration of qualitative and quantitative data, (3) consistency between components, (4) interpretation of integrated findings, and (5) adherence to quality criteria of each methodological strand.

### 2.4. Stage 4: Data Extraction

The extracted data encompassed bibliometric (e.g., authors, authorship, country and region of origin, year of publication) and research information (e.g., article type, journal type, title, purpose, outcomes, keywords, setting, subjects, and sample). A minimum of four authors collected and compared primary data. In the event of disagreement, consensus within the group was prioritized. Inter-rater reliability was evaluated using the Covidence application, which was recognized as an excellent tool for literature reviews [19,20]. Data reconciliation was also conducted through the Covidence application, ensuring proper management of the data. Following successful conversion to a spreadsheet, extracted data were also organized and managed in an Excel spreadsheet, allowing the team to systematically group studies by key characteristics (e.g., study design, setting, or population) and identify differences across studies. This approach facilitated both quantitative summaries (e.g., counts of studies by country, type, or sample size) and qualitative comparisons (e.g., thematic alignment of outcomes or purposes). For the thematic analysis, all qualitative data, including study purposes, outcomes, and key findings, were independently reviewed by at least two authors to generate preliminary codes. Codes were iteratively compared, refined, and reconciled to develop a consensus coding framework. Inter-coder reliability was assessed through discussion and by calculating percentage agreement for key variables, ensuring consistency across studies. Once consensus was achieved, codes were grouped into broader themes reflecting patterns in the literature, such as health management, social connectedness, emotional support, and technology adoption. Following extraction, coding, and thematic synthesis, all data and supporting spreadsheets were securely stored in institutional databases for further analysis.

### 2.5. Stage 5: Qualitative and Quantitative Meta Synthesis

Following data extraction, the review process involved a comprehensive analysis that distinguished between qualitative and quantitative methodologies. Each research design underwent individual syntheses, as outlined in the published framework [16], prior to the final mixed-methods synthesis. This process is predicated on the premise that both designs represented fundamentally different entities, necessitating separate analyses and interpretations of their respective findings. Studies employing both designs were differentiated, as the synthesis of qualitative data employs a methodology specifically tailored to its characteristics, while the synthesis of quantitative data necessitated an approach that is uniquely suited for its design. This segregation in the analyses was essential for accurately capturing and understanding the distinct contributions of each research design in understanding the effectiveness of smart speakers on the health outcomes of older adults.

### 2.6. Stage 6: Mixed-Methods Synthesis

The syntheses of the two distinct research designs have been subject to a comprehensive ‘mixed methods’ synthesis. This analysis integrated qualitative methods, such as meta summary, constant comparative analysis, and reciprocal translation of concepts [21], alongside quantitative findings derived from meta-analysis techniques. As stipulated in the framework, the synthesis of both research designs was conducted only after the qualitative and quantitative findings had been independently synthesized using methodologies distinctive to each. At this stage, the results were presented as a series of conclusions or recommendations, theoretical frameworks, or path analyses, depending on the nature of the relationship between the findings, whether they supported (confirmation), contradicted (refutation), or simply added to each other (complementary) [16]. Integrating the findings from the two research designs has provided a nuanced interpretation of the acceptability and effectiveness of smart speakers within the specific context of this review.

### 2.7. Ethics

There are no human participants, as the review uses data from previously published articles. Moreover, this study was deemed exempt from review by the University of Central Florida (STUDY00008064) and the Our Lady of Fatima University—Institutional Ethics Review Committee (2025-IERC-00608). This study has been registered at OSF (https://doi.org/10.17605/OSF.IO/M3C7J).

## 3. Results

### 3.1. Article Bibliometrics

A total of 20 articles focusing on the effect of using smart speakers on the healthcare of older adults were included in the bibliometric analysis. Table 2 presents the bibliometric data for the twenty (n = 20) articles reviewed. Most of these articles were authored by multiple contributors (n = 14; 70%) and published in health journals (n = 19; 95%) in 2021 (n = 6; 30%), mostly originating within the American region (n = 12; 60%). Most studies examined samples of healthy older adults (n = 15; 75%), typically consisting of 11–20 participants (n = 7; 35%), in community settings (n = 8; 40%), aligning with the aim of assessing smart speaker usability and health-related benefits in the lives of older individuals.

### 3.2. Article Purpose and Outcomes

This review comprised 20 articles that examined the use of smart speakers in the health and well-being of older adults. Two key areas were examined from the studies, namely the article’s purpose and outcomes that span the primary objectives and core results. Table 3 presents the research articles, including their corresponding authors, focus areas (physical, mental, and social health), quality checks based on MMAT parameters, relevance, purpose, and outcomes. To clearly distinguish emerging patterns, Table 4 presents thematized purposes and outcomes of publications on smart speakers for geriatric care. In terms of purpose, the studies were classified into five categories: (1) Health Management and Well-being, (2) Psychological and Emotional Support, (3) Social Connectedness, (4) Technology Adoption and Feasibility, (5) Usability and Design Development. Similarly, the resulting outcomes of each article were also categorized into five: (1) Improved Daily Living, (2) Positive Emotional and Social Effects, (3) Health Benefits, (4) Adoption Challenges, (5) Opportunities for Refinement. Based on the MMAT appraisal, eight studies (40%) achieved a score of 100%, indicating high methodological quality. Another eight studies (40%) were rated at 80%, reflecting moderate quality, while the remaining four studies (20%) scored 60%, suggesting areas of methodological limitation.

The thematic synthesis of findings shows outcomes on physical, mental, and social health, alongside barriers and opportunities for smart speaker use. Physical health outcomes were addressed through applications that promoted cognitive function, exercise, and self-management. Programs such as smart speaker–based metamemory training have improved memory and attention scores [30]. Feasibility studies have also demonstrated their potential for delivering physical activity interventions [12,34]. Smart speakers were also used for reminders and health information, leading to positive behavioral changes in some cases [29]. Despite these promising results, technical barriers such as Wi-Fi connectivity issues, difficulty phrasing commands, and system errors limited effectiveness and scalability [12]. In terms of mental health outcomes, smart speakers were consistently associated with reduced loneliness and depression among older adults, particularly those living alone [14,25]. Devices were also described as companions, contributing to emotional support and well-being [8,27]. However, the positive effects were moderated by frustrations with limited conversational depth, personalization, and concerns about privacy and security, which often tempered initial enthusiasm [13,23].

Additionally, social health outcomes highlighted how smart speakers enhance connectedness and reduce isolation. Older adults used them to maintain daily routines, access entertainment, and facilitate interactions with family or caregivers [7,10]. Devices were particularly valuable in care settings, where staff leveraged them to engage residents [28]. Despite this, evidence suggests that smart speakers complement rather than replace human-to-human interactions, serving more as facilitators of social contact than substitutes [35]. Barriers and design opportunities were consistently reported across the evidence base. Adoption was limited by usability challenges (e.g., phrasing commands, personalization), cost, and privacy/security concerns [13,23]. At the same time, studies highlighted design opportunities to enhance accessibility, trustworthiness, and age-friendliness [22,33]. Successful adoption often depended on external support from caregivers or staff, underscoring the need for training and user-centered design.

### 3.3. Mixed-Methods Outcomes

**Figure 2:**
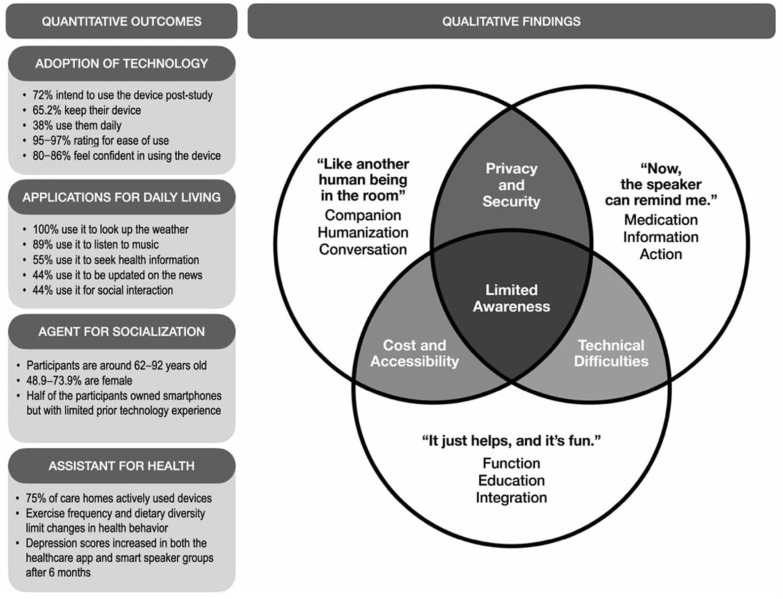
Quantitative and Qualitative Study Outcomes on Smart Speakers for Health and Well-being of Older Adults.

A mixed-methods review was conducted to systematically integrate quantitative and qualitative findings from the included studies. To summarize and link the data from the two research types mentioned, a mixed-methods joint display was employed. This tool facilitates data collection, which is used in planning, implementation, and the presentation of results [36]. Figure 2 presents a thematic analysis of the joint display of quantitative and qualitative study outcomes. In terms of the quantitative results, the findings were organized into categories, including the adoption of technology, applications for daily living, agents for socialization, and assistance for health. These findings highlighted high adoption rates (e.g., 72% of participants reported an intent to use and 65.2% chose to keep the device after the study), as well as frequent use for everyday tasks (e.g., 100% use for weather inquiries, 89% for music listening, 55% for seeking health information). In parallel, the qualitative findings were summarized into themes, including privacy and security, cost and accessibility, technical difficulties, and limited awareness. The three-circle Venn diagram shows areas of overlap in older adults’ use of smart speakers. The main barriers were grouped into privacy and security, cost and accessibility, and technical difficulties. Existing overlaps exacerbate these challenges, such as a lack of resources, which make technical issues more difficult to resolve and heighten privacy concerns. Alongside this, participants across studies also reported positive experiences, describing the device as “*like another human being in the room*”, noting that “*it just helps, and it’s fun*”, and valuing reminders that supported daily routines.

### 3.4. Keyword Co-Occurrence Network Analysis

**Figure 3:**
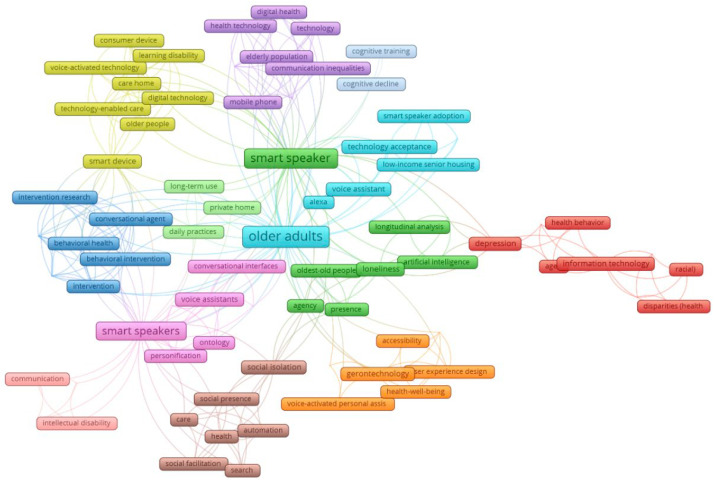
Article keywords: co-occurrence and topic clusters network map.

Figure 3 presents the co-occurrence network of article keywords, revealing eight thematic clusters from the review (Table 5) that demonstrate the characteristics of research on smart speakers for older adult health. The network revealed that “smart speakers”, “older adults”, “social isolation”, and “social presence”, among others, are some of the most frequently occurring keywords. Analysis of the literature revealed eight interrelated clusters highlighting the multidimensional role of voice-enabled technologies for older adults. These include technology and health equity, emphasizing access and reducing disparities; AI and social connection, focusing on communication and emotional support; technology-enabled care and support, targeting independent living and aging in place; and digital health and well-being, addressing physical, cognitive, and mental health outcomes. Additional clusters highlight voice assistant adoption and quality of life, accessible gerontechnology and design, human-like interaction in voice technology, and technology-assisted cognitive and behavioral interventions, reflecting research on usability, engagement, and functional support. Together, these clusters demonstrate that the field spans technological innovation, social support, health promotion, and design considerations for aging well.

## 4. Discussion

These mixed-methods review examined the effects of smart speakers on the health and well-being of older adults. Specifically, it aimed to synthesize findings that will substantiate the acceptability and effectiveness of smart speaker technology in achieving intended health outcomes among older adults. Given the increasing prevalence and benefits of this technology in daily life, this analysis has significant potential to provide valuable insights into the application of innovative technologies to address the specific needs of this population.

### 4.1. Article Bibliometrics

Most studies were published in health and computer science journals. Geriatric care is interdisciplinary, particularly in technology integration topics, such as smart speakers, which require not only clinical evaluation but also technical design expertise. To improve geriatric care, an interdisciplinary approach is necessary [37]. A collaborative approach is crucial for effective geriatric care, including the strategic use of technology. The finding demonstrates that research on smart speakers for geriatric care is a multidisciplinary endeavor. Furthermore, this result promotes interdisciplinary collaboration among gerontology researchers and other experts, facilitating the development of novel innovations and knowledge exchange.

Studies on smart speakers for older adults began in 2019, with peaks in 2021 and 2024. Researchers are acknowledging the potential impact of smart speakers on the older adult cohort. During 2021, older adults faced social isolation due to the COVID-19 pandemic, resulting in a surge in articles at that time. Furthermore, since its adoption during the pandemic, digital health is expected to be normalized by 2024. Social isolation among older adults was a standard issue during the pandemic [24,38]. In addition, studies on initial interaction with smart speakers [13] and further exploration of the topic [39] arose during the same year. Moreover, by 2024, the emergence of more qualitative and usability investigations was an indicator that research in the area had progressed into deeper contexts [8,10,24]. However, the number of published studies followed a wave-like pattern from 2019 to 2025, with crests in 2021 and 2024. The year 2021 reflects the need for interventions during the pandemic, while 2024 marks a period of maturation in the field. Currently, the findings indicate that researchers and funders face a challenge in sustaining research in the field beyond crises, such as the pandemic. There is a need to sustain scholarship on the use of smart speakers among older adults. The historical and technological context in which these studies were conducted may also have influenced their outcomes. Early research, typically published before 2019, often reflected limited device capabilities, lower levels of digital literacy among older adults, and initial skepticism toward voice-activated technologies. In contrast, more recent studies, particularly those conducted during or after the COVID-19 pandemic, were shaped by accelerated digital adoption, heightened reliance on remote communication, and growing familiarity with AI-driven devices [40]. These contextual differences likely affected participants’ attitudes, patterns of use, and perceived usefulness of smart speakers.

The American region has contributed the most articles of any area of the world. Most of the articles featured smart speakers from American companies, such as Amazon and Google, which can explain the region’s dominance in the field. The United States has initiated the commercialization of smart speakers via large companies such as Amazon and Google. Based on a bibliometric analysis concerning technology for aging, the USA leads in the number of articles, demonstrating its role as a forerunner in technological innovation for older adults [41]. In addition, the country leads in the number of publications on technology acceptance among older adults [42], further indicating its pace compared to other regions. This result implies that innovation is concentrated in the American region, and technology-based health solutions for older adults are American-centric in design. This finding poses another challenge: there is a clear need to co-design technologies for older adults from diverse cultural contexts and to ensure that smart speakers can adapt to the needs of those outside America.

Most articles employed an 11 to 20 sample size consisting of normal older adults, while a few mentioned including non-healthy participants, specifically those with physical disabilities. Potential reasons include that studies are still in the pilot stage, which may be due to ethical issues surrounding studies involving older adults as vulnerable participants [43]. One barrier to recruiting older adults into trials is health issues, as comorbidities negatively affect study retention [44]. However, the generalizability of studies utilizing only healthy older adults is limited. This creates a knowledge gap, which underrepresents those older adults with comorbidities [45].

Additionally, this also demonstrates a bias towards the healthy population [46]. Moreover, researchers are encouraged to be more inclusive when recruiting older adult participants to ensure proper representation and demonstrate the principle of justice [47,48]. Due to these factors, it is necessary to examine the use of smart speakers among older adults with health challenges.

Community and home settings are common sites of research in these studies. Consumer devices, such as smart speakers, are created for everyday home use. To see authentic usage patterns among older adult participants, home and community sites are preferred. Additionally, the current features of smart speakers (e.g., grocery lists) make them well-suited for home use. For instance, many studies on technologies for older adults [49,50], aside from smart speakers, have been conducted in participants’ homes to empower aging in place, a principle highlighted in this finding. Additionally, this demonstrates the ecological validity of the studies, as most are conducted in the communities of older adults. However, generalizability is also limited in this aspect because it applies only to those in clinical settings and nursing homes. It is recommended that future researchers expand their research to include clinical and institutional settings, thereby catering to all types of participants and increasing diversity in the field, as smart speakers have high potential for smart home integration.

### 4.2. Article Purpose and Outcomes

The review of article purposes and outcomes revealed that the objectives of the studies highlighted the multifaceted role of smart speakers in geriatric care, encompassing: (1) health management and well-being, (2) psychological and emotional support, (3) social connectedness, (4) technology adoption and feasibility, as well as (5) usability and design development. This may be linked to the diverse purposes of the reviewed studies that reflect the multidimensional needs of older adults and the multifunctionality of smart speakers. In geriatric medicine, older adults require assessments that encompass physical, mental, functional, and social domains, among others, to provide holistic care that goes beyond mere medical evaluation. This is embodied in the concept of Comprehensive Geriatric Assessment (CGA), a diagnostic process that encompasses medical, psychosocial, and functional domains to understand the needs of older adults [51,52]. The multiple functions that smart speakers are capable of providing thus address this, wherein in care homes, smart speakers were shown to be used for diverse purposes including music, news and weather updates, quizzes and games, reminders, and video calls, illustrating their versatility in supporting emotional, social, informational, and routine needs [28]. It was also observed that smart speakers can provide significant benefits for older adults, including increased convenience and improved quality of life; however, to promote maintainable use behaviors, designers and developers should consider more about the technology use contexts and the specific needs and preferences of older adults when designing these devices [10]. The multifaceted purposes identified across articles can evolve into versatile tools for geriatric care, as remote health assessment is a potential area of future research [53].

In addition to the objectives, the articles’ outcomes demonstrated improved daily living, positive social and emotional effects, and notable health benefits, while also revealing persistent adoption challenges and underscoring opportunities for refinement. While smart speakers are primarily designed to support the activities of daily living (ADLs) of the general population, there remains substantial room for improvement in the technology, especially in older adult healthcare [54]. Findings from care home implementations demonstrated that older adults experienced enjoyment, relaxation, and companionship, which were noted as positive emotional responses when using smart speakers. It was also noted that voice-activated technologies are easy to use and promote interaction, as they offer multifaceted benefits to residents and staff [28]. While these results underscore broader gains, various factors—such as age-related issues (e.g., low digital literacy, sensory limitations) and device-related issues (e.g., privacy concerns, usability issues, cost)—contribute to adoption challenges. Long-term use of smart speakers faces challenges, including limited understanding of their capabilities, insufficient training, and skepticism about their essential role in aging in place. For instance, older adults were reported to experience difficulty in understanding the full spectrum of functionality and features of the Alexa device, as they received relatively little initial and ongoing training and support in using it [31]. These persistent hurdles in adoption suggest the need for structured training programs, user-friendly guides, and caregiver support to ensure sustained use. Smart speakers designed with Human-Centered Design (HCD) principles could better align with geriatric care by incorporating features that support sensory, memory, and physical needs, thereby helping maintain independence and enhance daily living experiences.

These findings suggest specific actions to translate research into practice. Policymakers could implement subsidy programs to make smart speakers affordable for older adults, establish privacy and usability standards for voice-activated devices, and integrate these technologies into government-supported community centers or residential care programs. Designers and developers should create devices with large, clearly labeled buttons, simplified voice commands, context-sensitive prompts, and customizable reminder systems that address medication schedules, exercise, and social interactions. Clinicians and caregivers can actively incorporate smart speakers into daily care routines, for example, using devices to deliver medication reminders, facilitate virtual family calls, or guide cognitive and physical exercises, while providing brief, hands-on training sessions to ensure older adults understand all key functions and use them consistently.

Although these findings yield clear directions for practice and policy, the degree to which they can be generalized depends on the methodological rigor of the studies reviewed. In interpreting these findings, it is also significant to acknowledge the heterogeneity in research quality across the included studies. While several investigations demonstrated methodological rigor through clearly articulated mixed-methods designs, validated tools, and systematic integration of qualitative and quantitative data, others demonstrated lower methodological quality. Common limitations included small or non-representative samples, unclear sampling strategies, and minimal detail on data integration procedures between quantitative and qualitative components. Some qualitative studies lacked transparency in coding or trustworthiness procedures (e.g., triangulation, member checking). At the same time, specific papers relied on cross-sectional or exploratory designs without control groups or standardized outcome measures. These limitations constrain the strength and comparability of the evidence base and may partially account for inconsistencies in reported outcomes regarding usability, adoption, and well-being. Future mixed-methods research should employ rigorous integration frameworks and validated assessment tools to enhance the reliability of findings on smart speaker use among older adults.

### 4.3. Mixed-Methods Outcomes

#### 4.3.1. Adoption of Technology

The adoption of technology across the reviewed articles yielded quantitative and qualitative results demonstrating how smart speakers shape users’ experiences, perceptions, and health and well-being. The results indicate that older adults have a high acceptance rate and a positive outlook toward smart speakers. Previous studies reported a high acceptance rate of smart speakers among contacted care homes [26]. It was also shown that 38% of older adults used devices daily, with another 38% using them several times per week [7]. Regarding continued use, older adults expressed a desire to keep using the devices after study completion, suggesting not only short-term feasibility but also longer-term acceptability [7]. Adoption was also shown to be influenced by factors such as ease of use, user confidence, and placement flexibility. Ease of use was consistently rated high (95–97%), indicating that older adult participants found the devices intuitive [28], with most feeling confident in their ability to operate them (80–86%). These statistics underscore the value of user-friendly design [25]. Finally, user placement revealed that 65% of devices were positioned in communal areas to facilitate regular use [28]. The identified findings suggest that user capabilities and environments heavily influence technology adoption [55], highlighting the importance of pairing technological interventions with environmental and social supports to foster sustained use in care settings.

Despite the largely favorable quantitative results, the qualitative analysis revealed that older adults’ adoption of smart speakers was characterized by limited awareness of their full capabilities, along with concerns about privacy, security, technical issues, cost, and accessibility (Figure 2). This observation may be possibly attributed to a gap that reflects the broader mismatch between the barriers of smart technology and the everyday realities of older adults, including financial, technical, and social circumstances [56,57]. Privacy and data security remain among the most persistent ethical barriers to the widespread adoption of smart speakers in geriatric contexts. To address these challenges, researchers recommend user-centered privacy designs that combine transparency, consent management, and adaptive privacy settings. For instance, providing context-aware explanations (i.e., why the device activates, where data is stored, and how it’s used) can foster trust and informed use [58].

Older adults often face difficulties using online platforms due to non-age-friendly interfaces and limited assistance features, which can lead to lower participation and distrust in online healthcare. Concerns about privacy, technology anxiety, and low digital literacy exacerbate these issues, as older adults fear the accidental disclosure of personal information [59], as portrayed in media reports on data breaches, hacking, and similar instances (An et al., 2025) [60]. Especially for older adults with fixed incomes [60], cost concerns have also been identified as a major barrier to adopting smart technology, due to the costs of enabling and maintaining the continuous operation of complete and pervasive devices [61]. Taken together, the quantitative findings of high ease-of-use ratings (95–97%) and confidence levels (80–86%) are consistent with qualitative reports describing the devices as “helpful” and “fun.” This convergence suggests that perceived simplicity and positive affective experiences are key determinants of sustained engagement among older adults. However, the divergence between high intention to use (72%) and limited awareness, as identified qualitatively, indicates that familiarity with the device’s full range of functions remains low. These findings suggest that while smart speakers have potential for supporting the daily living of older adults, their successful adoption depends on addressing practical barriers. It would be beneficial for future research to emphasize reducing technical complexity and the role of care providers in training and awareness-building, to increase confidence and ease of use.

#### 4.3.2. Applications for Daily Living

Quantitative and qualitative analyses demonstrated that older adults utilize smart speakers in various ways, including checking the weather, providing reminders, accessing entertainment, making calls, engaging in conversations, seeking information, and tracking time. This finding indicates that using smart speakers is not only convenient for older adults but also extends to the intersection of various health domains. Technology has been utilized to support the well-being and independence of older adults, expanding to various technologies integrated into geriatric care [61]. These technologies are currently employed in daily living and have multiple health applications [62,63]. For instance, various studies demonstrated that smart speakers are used by older adults daily [10] for physical [22,23], mental [7,32], and social health [8,31]. Due to this, it can be inferred that smart speakers are already integrated into geriatric care. This indicates that it presents an opportunity to develop a more structured health support system, utilizing smart speakers, across various health domains for older adults.

However, based on qualitative analyses, issues such as command structures, wake words, and other technical requirements make it difficult for older adult users to utilize smart speakers. As evidenced by this result, there is a mismatch between the technological competency and literacy of older adults and the technological requirements of smart speakers. For example, a qualitative study [64] revealed that low-income older adults face challenges, including usability issues and difficulties with learning technology, when using voice user interfaces or smart speakers. Several barriers were identified when using smart speakers, including a lack of technological competency and difficulties issuing commands [30]. Quantitative indicators also show that frequent device use for tasks such as weather updates and entertainment align with qualitative narratives portraying smart speakers as tools for convenience and independence. This convergence illustrates that routine, low-effort interactions serve as gateways to technology acceptance and that smart speakers, even though seen as tools to make users’ lives easier, can become a hindrance in everyday life. Developers of smart speakers are encouraged to increase the devices’ accessibility to bridge the gap in older adults’ capabilities.

Based on some qualitative analyses, an additional advantage of smart speakers is that it is portable and can integrate with other devices or appliances. This indicates that smart speakers are not just stand-alone assistance devices but also connect various living environments, making them ideal tools for supporting the health and well-being of older adults. For instance, smart speakers can be set up anywhere, based on a user’s daily habits [10], and can be integrated into smart devices [65]. Since integration and portability are the cornerstones of smart speakers, these devices can serve as personal central hubs for aging in place, enabling older adults to manage their health and safety and maintain their independence.

#### 4.3.3. Agent of Socialization

Research participants included individuals from lower socioeconomic backgrounds, which contrasts with the majority of existing literature. Previous studies have consistently reported that individuals with lower income levels are less likely to participate in health research, often citing barriers such as financial costs, travel difficulties, and the need to prioritize work or employment obligations [66]. The inclusion of participants from these backgrounds in the articles underscores the significance of contextual factors, particularly the home-based setting of the studies and the representation of retired and non-working individuals. The findings also suggest that device ownership of older adults does not necessarily translate into digital competency. For older adults, ownership alone is insufficient to enhance digital skills; rather, competency depends heavily on the availability of support, guidance, and personalized learning opportunities, which can improve both technological use and meaningful digital engagement [67]. Analysis of participant profiles also reveals gendered differences in adoption and engagement, as the article’s primary participants were predominantly female. This could be attributed to males demonstrating greater suspicion, distrust, and tendencies toward conspiratorial thinking regarding the research process compared to females [68]. Enhancing the representation of participants and studies through balanced inclusion is therefore essential to generate more comprehensive insights concerning the social and emotional effects of smart speaker use.

Qualitative analyses further demonstrate how smart speakers can extend beyond their utility and function as virtual companions. The natural, conversational features of smart speakers are often treated by their users as social interactions rather than commands. The mechanical voice of virtual agents makes their respectful language seem unnatural, which leads to a low reported social presence, showing a preference for agents with higher social presence over inanimate tools [69,70]. These interactions, which users consider social rather than functional, lead them to anthropomorphize the device and attribute human qualities to it, often describing it as their “best friend” or “like another human being in the room”. Research also shows that older adults report higher satisfaction with companion robots that display greater levels of anthropomorphism, with anthropomorphism exerting a significant positive effect on user satisfaction [71]. For older adults who live alone and experience social isolation, the companionship they derive from anthropomorphized objects is understandable. As individuals age, cognitive, emotional, and physical changes may influence their attachment to objects and affect how they view their possessions over time [72]. The overlap between quantitative indicators of use and qualitative reports of emotional attachment reflects that social connectedness, not technological sophistication, drives continued adoption in socially isolated groups.

#### 4.3.4. Assistant for Health

The review found that smart speakers have a positive impact on users’ physical and mental health, particularly in structured settings such as care homes. Quantitative analysis emphasized that older adults’ use of smart speakers demonstrated both benefits and limitations across health-related outcomes (Figure 2). Intervention effectiveness varied, with physical activity programs showing initial engagement but declining usage over time; however, participants maintained their intention to continue [12]. In home care settings, engagement was promising, with 75% of participating care homes actively using the devices [28]. However, measurable health behavior changes were limited, with no significant improvements observed in exercise frequency or dietary diversity [29]. Mental health outcomes were similarly mixed, as depression scores increased in both healthcare app and smart speaker groups after six months, with no significant differences between them [30]. On the other hand, the outcomes of loneliness varied with frequency of use, highlighting the importance of sustained and meaningful engagement [14].

In terms of its corresponding qualitative findings, older adults’ experiences with smart speakers revealed the devices’ usefulness as health coaches, capable of offering medication reminders, providing information, and assisting with daily routines. Medication non-adherence is a major global health issue, linked to increased morbidity, mortality, and healthcare costs, with older adults particularly at risk due to polypharmacy, multimorbidity, and reduced autonomy [73]. Within this context, the ease of use and accessibility of smart speaker devices may be especially valuable, as voice interaction removes the need for heavy technical skills, typing, or screen navigation, making them suitable for older adults with limited digital literacy, vision impairment, or mobility challenges [13]. This functionality not only addresses practical barriers to technology use but also enables older adults to engage more consistently in health-promoting routines, reinforcing the role of smart speakers as supportive tools for daily self-management. Evidence suggests that while smart speakers are acceptable and feasible for older adults, their long-term effectiveness in driving consistent physical or psychological improvements remains uncertain. While smart speakers’ ease of use via voice interaction makes them suitable for older adults with limited technological and health capabilities, mixed quantitative results (e.g., variable mental health outcomes) suggest that simply providing smart speakers may not be sufficient to achieve meaningful health improvements. [28,74,75]. Quantitative measures revealed modest or inconsistent improvements in physical and mental health, but qualitative accounts provided explanatory depth: older adults described the speaker as a reliable reminder system and supportive “coach.” This indicates that while the statistical impact may be limited, the subjective sense of empowerment and structure derived from voice-based assistance contributes to perceived well-being.

Beyond their standalone functions, smart speakers also have the potential to complement other emerging geriatric technologies, creating synergistic systems that enhance older adults’ health, safety, and social well-being [10]. When integrated with wearable health trackers, smart home sensors, or telehealth platforms, smart speakers can serve as intuitive voice-based hubs that gather, communicate, and respond to real-time health data [76]. For instance, integration with fall-detection devices or medication dispensers could allow voice alerts and automated emergency calls, while connections with remote monitoring systems could facilitate routine health check-ins and adherence tracking. Combining these technologies may reduce caregiver burden, support early intervention, and promote more responsive and personalized care environments.

To further advance the field of smart speaker integration in geriatric care, future research should consider linking current findings to broader trends in digital health, health education, and AI-driven tools. mHealth interventions have been shown to improve health literacy among older adults [77], while AI technologies, including virtual assistants and robotics, are being explored in long-term care settings [78,79]. Integrating smart speakers with such tools could enhance personalized care, engagement, and aging-in-place strategies, positioning them as part of a broader digital ecosystem for older adult care.

### 4.4. Keyword Co-Occurrence Network Analysis

The results of the keyword network analysis revealed eight major thematic clusters: (1) Health Equity and Inclusion, (2) Artificial Intelligence and Social Health, (3) Digital Home Healthcare, (4) Digital Health and Well-being, (5) Digital Health Agents and Quality of Life, (6) Health Technology and Usability, (7) Humanized Technology and Representation, (8) Health Behavior and Cognition. Reflecting both the technological and psychosocial dimensions of this emerging field, the first cluster highlights how access, affordability, and digital literacy influence smart speaker use among older adults, emphasizing the importance of inclusive design and equitable access to prevent the widening of the digital divide [80]. Similarly, the second cluster underscores how smart speakers foster social connection and reduce loneliness among older adults, demonstrating that through enabling conversational engagement, voice assistants are increasingly viewed as tools for emotional support; however, ethical concerns remain regarding the authenticity of interaction and potential emotional dependency on AI. In this regard, Cluster 3 focuses on the role of smart speakers in remote monitoring, care coordination, and aging in place, highlighting that smart speakers can support caregivers and enhance independent living, but that challenges persist around privacy, data security, and autonomy in technology-assisted care environments [81].

The fourth, fifth, and eighth clusters are observed to focus on improving mental well-being, and hence, quality of life. Across literature, smart speakers are positioned within digital health ecosystems that support physical and mental well-being, as these can assist in self-management, telehealth, and preventive care [29]. Nonetheless, further clinical validation and long-term evaluation are needed to confirm their sustained health impacts. These clusters assess factors influencing technology adoption, including usability, trust, and perceived usefulness. Consistent with TAM and UTAUT models, adoption is shown to be linked to improved autonomy and life satisfaction, underscoring the importance of ensuring age-friendly design to promote positive user experiences [82]. In terms of design, Cluster 6, anchored in user-centered and participatory design, calls for co-creation with older adults to enhance accessibility and engagement. Smart speakers that are intuitive and adaptable can foster empowerment and aging well, as demonstrated in Cluster 7, which ultimately elaborates on creating natural, empathetic, and engaging interactions through voice technology and human-like qualities that increase comfort and trust [83].

The results of this review highlight several theoretical and practical implications. Thematically, the clusters reveal an interdisciplinary integration of gerontology, digital health, and human–computer interaction, suggesting that future frameworks should move beyond usability and adoption to also consider emotional engagement, ethical perception, and contextual equity in technology use among older adults. Future research should adopt longitudinal and mixed-methods approaches to assess sustained health outcomes, cognitive benefits, and emotional impacts of smart speaker use. Comparative and cross-cultural studies are also needed to understand how sociocultural contexts shape adoption and engagement.

## 5. Conclusions

This mixed-methods review examines published articles on health interventions using smart speakers for older adults. It aimed to map the characteristics of existing studies and assess the current state of this domain using both quantitative and qualitative articles for a comprehensive review. A thorough literature search was conducted across five major databases (Scopus, Web of Science, PubMed, ProQuest, and IEEE Xplore) to identify relevant articles. Through systematic screening and quality evaluation using the Mixed-Methods Appraisal Tool, 20 studies were selected for final analysis. The research team extracted key information, including publication details, intervention goals, health-related outcomes, and device characteristics, to identify thematic patterns.

The results further revealed that most articles originated from the American region and featured smart speakers from American companies, such as Amazon and Google. An analysis of the articles resulted in the following purposes being determined: (1) Health Management and Well-being, (2) Psychological and Emotional Support, (3) Social Connectedness, (4) Technology Adoption and Feasibility, and (5) Usability and Design Development; whereas the outcomes reflected corresponding categories: (1) Improved Daily Living, (2) Positive Emotional and Social Effects, (3) Health Benefits, (4) Adoption Challenges, and (5) Opportunities for Refinement.

The older adults’ adoption of smart speakers was marked by limited awareness of their full capabilities, along with concerns about privacy, security, technical issues, cost, and accessibility. Smart speakers are being used to support older adults’ daily living, highlighting their growing role in geriatric care. Their portability and ability to integrate with other devices make them valuable hubs for aging in place, highlighting opportunities to design more structured and accessible health support systems while maintaining independence for older adults. Participants from lower socioeconomic backgrounds were also included, although the findings showed that device ownership did not guarantee digital competency. Most participants were female, while males were shown to be less engaged due to greater distrust in research. Qualitative analyses also indicated that older adults often anthropomorphized smart speakers, treating them as companions, especially among those experiencing social isolation. As health assistants, smart speakers have shown positive effects on both physical and mental health, particularly in care homes, although the quantitative results have been mixed. Engagement in physical activity programs declined over time, which limited the effectiveness of health behavior changes. Mental health outcomes showed no significant improvement, while loneliness outcomes varied by frequency of use. On the other hand, qualitative findings highlighted the usefulness of smart speakers as health coaches, supporting medication adherence and daily routines. Voice interaction reduced usage barriers for older adults; however, their long-term effectiveness remained uncertain without integration into formal care systems.

Ultimately, evidence suggests that smart speakers have strong potential to enhance the physical, mental, and social well-being of older adults. By supporting independence, daily routines, emotional health, and social connectedness, these devices can serve as versatile tools for physical activity, companionship, and entertainment. Existing findings underscore the need for future research to develop smart speaker integration in geriatric care, with particular emphasis on vulnerable elderly populations, ensuring accessibility, user support, and evidence-based interventions that maximize benefits and promote healthy aging. Future systematic reviews may also benefit from integrating gray literature sources, as program evaluations, government reports, and unpublished theses, to capture practical and policy-level applications of smart speaker use among older adults, particularly in community and care-home settings. Researchers should consider using structured mixed-methods synthesis frameworks (e.g., framework synthesis, meta-analysis, meta-aggregation, convergent designs) to improve rigor and clarity in integrating qualitative and quantitative findings. Where feasible, statistical analyses, including effect sizes and subgroup comparisons (e.g., by device type, study design, or outcome domain), can further strengthen inferences and support evidence-based recommendations.

## Figures and Tables

**Table 1:** Database Search Strategy.

Database	Search Strategy	Articles
Scopus	(TITLE-ABS-KEY (“older adult*” AND “smart speaker*” OR “smart” AND (“voice” OR “audio”)) AND TITLE-ABS-KEY (“health” OR “well-being” OR “loneliness” OR “wellness” OR “behavior” OR “emotion” OR “depression” OR “social” OR “connected*” OR “isolation” OR “physical”)) AND (LIMIT-TO (DOCTYPE, “ar”))	56
Web of Science	(TS = (“older adult*” AND (“smart speaker*” OR (“smart” AND (“voice” OR “audio”))) AND (“health” OR “well-being” OR “loneliness” OR “wellness” OR “behavior” OR “emotion” OR “depression” OR “social” OR “connected*” OR “isolation” OR “physical”))) AND ((DT == (“ARTICLE”) AND LA == (“ENGLISH”) AND SJ == (“BEHAVIORAL SCIENCES” OR “MEDICAL INFORMATICS” OR “PSYCHOLOGY” OR “HEALTH CARE SCIENCES SERVICES” OR “RESEARCH EXPERIMENTAL MEDICINE” OR “MEDICAL LABORATORY TECHNOLOGY” OR “COMPUTER SCIENCE” OR “SOCIAL ISSUES”)) NOT (SILOID == (“PPRN”)))	77
PubMed	(“older adult”[tiab] OR “older adults”[tiab] OR “aging”[tiab]) AND ((“smart speaker”[tiab] OR “smart speakers”[tiab]) OR (“smart”[tiab] AND (“voice”[tiab] OR “audio”[tiab]))) AND (“health”[tiab] OR “well-being”[tiab] OR “loneliness”[tiab] OR “wellness”[tiab] OR “behavior”[tiab] OR “emotion”[tiab] OR “depression”[tiab] OR “social”[tiab] OR “connected”[tiab] OR “isolation”[tiab] OR “physical” [tiab]) AND ((ffrft[Filter]) AND (fft[Filter]) AND (english[Filter]))	43
ProQuest	((TI((“older adult” OR “older adults”) OR aging) OR AB((“older adult” OR “older adults”) OR aging) OR SU((“older adult” OR “older adults”) OR aging)) AND (TI(“smart speaker*” OR (smart AND (voice OR audio))) OR AB(“smart speaker*” OR (smart AND (voice OR audio))) OR SU(“smart speaker*” OR (smart AND (voice OR audio)))) AND (TI(health OR “well-being” OR loneliness OR wellness OR behavior OR emotion OR depression OR social OR connected* OR isolation OR physical) OR AB(health OR “well-being” OR loneliness OR wellness OR behavior OR emotion OR depression OR social OR connected* OR isolation OR physical) OR SU(health OR “well-being” OR loneliness OR wellness OR behavior OR emotion OR depression OR social OR connected* OR isolation OR physical))) AND LA(English) AND STYPE(“Scholarly Journals”)	73
IEEE	(“older adult*” OR “older adults”) AND (“smart speaker*” OR (smart AND (voice OR audio))) AND (health OR “well-being” OR loneliness OR wellness OR behavior OR emotion OR depression OR social OR connected* OR isolation OR physical)	51
	Total	300

**Table 2:** Article Bibliometrics.

Article Attributes	f	%
Authorship		
Single	2	10.00
Double	4	20.00
Multiple	14	70.00
Type		
Qualitative	11	55.00
Quantitative	5	25.00
Mixed	4	20.00
Research Area		
Health Science	8	40.00
Computer Science	0	0.00
Both	12	60.00
Journal Publication		
Health	19	95.00
Technology	1	5.00
Year		
2019	1	5.00
2020	1	5.00
2021	6	30.00
2022	4	20.00
2023	2	10.00
2024	5	25.00
2025	1	5.00
Region		
America	12	60.00
European	3	15.00
Southeast Asia	0	0.00
Western Pacific	5	25.00
African	0	0.00
East Mediterranean	0	0.00
Older Adult Sample		
Normal	15	75.00
Not normal *	5	25.00
Not mentioned	0	0.00
Sample Size (M = 51.80; SD = 71.16)		
0–10	3	15.00
11–20	7	35.00
21–30	2	10.00
31–40	2	10.00
41–50	1	5.00
50 and above	5	25.00
Environment		
Home	7	35.00
Community	8	40.00
Nursing home	4	20.00
Physical rehabilitation	1	5.00
Cognitive rehabilitation	0	0.00
Laboratory	0	0.00
Not mentioned	0	0.00

* Older adults with health conditions (diabetes or physical disabilities).

**Table 3:** Research Focus, Quality Check, and Purpose and Outcomes.

No.	Research	Focus ^A^	QC ^B^	R ^C^	Purpose	Outcomes
1	Chen et al. (2021) [22]	★●▲	100%	5	To identify barriers and design opportunities for using voice-based intelligent virtual assistants (IVAs) to improve healthcare management and quality of life among older adults	Older adults face multiple barriers in managing health and daily life, leading to design opportunities for more accessible voice-based assistants.
2	Astell & Clayton (2024) [8]	●▲	100%	5	To explore how older adults experience Amazon Alexa in their homes, particularly as a social presence that may reduce loneliness and support daily living.	Older adults perceived Alexa as a social companion that could ease loneliness and provide emotional support, though limitations in conversational ability and personalization restricted its effectiveness.
3	Chang et al. (2024) [10]	●▲	100%	4	To advance not only the development of age-friendly technologies but also the broader discourse on how technology can enhance the well-being of older adults.	Older adults successfully integrated smart speakers into their daily routines, primarily for entertainment, reminders, and information, which supported mental engagement and social connectedness, though limitations in personalization and advanced interaction restricted broader use.
4	Choi et al. (2021) [23]	★●▲	80%	4	To assess older adults’ perceptions of internet-of-things smart home devices over the course of a 2-month feasibility study.	Older adults showed favorable perceptions of IoT smart home technologies, recognizing their potential for health management and independence, but adoption was moderated by privacy, reliability, and cost concerns.
5	Chung et al. (2024) [24]	★▲	80%	2	To explore the relationship between smart speaker/ICT use and social connectedness, loneliness, and isolation in low-income older adults during the pandemic.	Smart speaker and ICT use were linked to greater social connectedness and reduced loneliness/isolation in older adults during COVID-19.
6	Chung et al. (2021) [25]	★	60%	2	To examine residents’ attitudes and perceptions toward smart speakers before and after use	Acceptance of smart speakers to perform basic tasks for daily living such as listening to music, time reminders, weather and news.
7	Corbett et al. (2023) [26]	★●▲	100%	3	To evaluate the feasibility of using voice-activated virtual home assistants (VHAs, i.e., Amazon Echo “Alexa” devices) with older adults participating in the Program for All-Inclusive Care of the Elderly (PACE)	Participants enjoyed the use of voice-activated virtual home assistants and PACE staff were enthusiastic about its potential.
8	Da Costa et al. (2024) [27]	★	100%	2	To evaluate the experiences and emotional response of older adults with type 2 diabetes regarding the use of virtual assistant device	Results highlight the positive emotional responses and strong sense of humanization expressed by elderly individuals with diabetes toward the virtual assistant device.
9	Edwards et al. (2021) [28]	★▲	80%	5	To explore if and how the devices were being used, the barriers to their implementation, and their potential benefits.	Implementation in care homes was possible and that smart speakers had multifaceted benefits for residents and staff.
10	Kim (2023) [29]	★	80%	5	To identify the effects of applying information and communication technologies (ICT) to the health management of older adults	Showed a positive health status and behavioral changes at post-evaluation but no reduced depression was observed
11	Kim et al. (2021) [30]	●	80%	5	To develop a smart speaker–based metamemory training (MMT) program and evaluate the efficacy of the program in older adults without cognitive impairment	The training group showed significant increases in the delayed free recall, digit span forward, digit span backward, and fluency test scores compared with the control group.
12	Kim (2021) [13]	●	80%	3	To investigate how older adults experience and respond to a voice assistant when they first interact with it.	Initial interactions with voice assistants were seen as simple and convenient—often used for health questions or music streaming and ending with polite responses—but follow-up experiences were largely negative due to difficulties in phrasing commands, misunderstandings of system operations, and concerns about privacy, security, and cost.
13	McCloud et al. (2022) [7]	●▲	60%	3	To investigate the feasibility of using smart speakers to improve the health and well-being of low-SEP older adults.	Most participants used the speaker regularly, found it useful, wanted to continue using it, and valued its role in providing companionship and connection, though health-related use was limited and initial challenges in framing questions were resolved with staff support.
14	Orlofsky & Wozniak (2022) [31]	●▲	100%	1	To understand how older adults actually utilize the Alexa VAPA in their daily lives, how they perceive their experience with the Alexa VAPA device, and the value that it brings to their overall aging experience and their ability to age in place.	Findings suggest that older adults, with limited training and support, mainly used Alexa for convenient but non-essential features, and did not view it as critical to aging in place.
15	Park & Kim (2022) [14]	●	60%	3	To examine whether frequent AI speaker use reduces depression and loneliness among older South Korean adults living alone compared to intermittent use.	AI-based smart speaker use was linked to reduced depression and loneliness in older adults living alone, with significant improvements in depression for both frequent and intermittent users and decreased loneliness among frequent users, highlighting its potential role in future psychological interventions.
16	Pradhan et al. (2019) [32]	●▲	100%	2	To understand how older adults use and perceive smart speakers	Voice assistants are categorized as “human-like” or “object-like”, participants fluidly move between the two categories based on factors specific to the characteristics of the voice assistant (such as nature of interaction) or the user (desire for social contact or affiliation), along with the location and moment of interaction.
17	Pradhan et al. (2020) [33]	●▲	100%	1	To understand how older adults who use technology infrequently perceive and use voice-based conversational interaction,	Older adults often used the device to access online information, particularly health-related content, highlighting the need to address concerns about credibility in voice-only interfaces. Although many were initially eager to use memory support features like reminders, actual use was far lower than expected.
18	Quinn et al. (2024) [12]	★	80%	2	To evaluate the feasibility and acceptability of using smart speakers to deliver a physical activity program to enhance older adults’ physical well-being.	Smart speakers were considered acceptable and highly usable for delivering physical activity programs, with participants appreciating the voice interface and daily task integration, though feasibility was hindered by technical issues such as Wi-Fi connectivity and command phrasing that required additional support.
19	Kurokawa et al. (2023) [34]	★	60%	2	To determine how smart speaker applications can be designed to promote continuous exercise and healthy aging in older adults.	The recommendation feature showed promise in promoting continuous exercise and improving self-efficacy, while the stamp feature was less effective; overall, the app was feasible but requires further refinement and testing with larger, more diverse samples.
20	Soubutts et al. (2022) [35]	▲	80%	4	To qualitatively examine how Amazon Echo supports the health, care, and well-being of socially isolated older adults during a pandemic.	Amazon Echo benefits the health and well-being of the participants; however, existing human-to-human social interactions are facilitated, rather than human-to-agent interactions.

^A^ Focus—★ = Physical, ● = Mental, ▲ = Social; ^B^ QC = Quality Check: 0–100% (0—poor; 100—excellent); ^C^ R = Relevance: 0–5 (0—not relevant; 5—highly relevant).

**Table 4:** Thematized Purposes and Outcomes.

Purpose		Outcomes	
Themes	Studies	Themes	Studies
Health Management and Well-being	[10,14,22,27,29,34]	Improved Daily Living	[10,25,26,28,33]
Psychological and Emotional Support	[7,8,14,25,27,35]	Positive Emotional and Social Effects	[7,8,10,14,25,27,32,35]
Social Connectedness	[8,25,35]	Health Benefits	[12,23,29,30,34]
Technology Adoption and Feasibility	[7,12,13,23,26,28,31]	Adoption Challenges	[10,12,13,22,23,26,31,33,35]
Usability and Design Development	[10,13,22,28,30,32,33,34]	Opportunities for Refinement	[22,28,34]

**Table 5:** Smart speakers for older adults: article keywords clustered by theme.

Cluster	Keywords	Main Themes	Description
1	Aged, Depression, Disparities (Health), Focus Groups, Health Behavior, Information Technology, Internet of Things, Racial, Social Well-Being	Health Equity and Inclusion	Articles examining the application of health technology to older adults
2	Agency, Artificial Intelligence, Loneliness, Longitudinal Analysis, Older Adult, Oldest-Old People, Presence, Smart Speaker, Automation, Care, Health, Search, Social Facilitation, Social Isolation, Social Presence	Artificial Intelligence and Social Health	Articles exploring Artificial Intelligence in relation to the social needs of older adults.
3	Care Home, Consumer Device, Digital Technology, Learning Disability, Older People, Smart Device, Technology-Enabled Car, Voice-Activated Technology, Daily Practices, Long-Term Use, Private Home	Digital Home Healthcare	Articles examining the use of digital home technology in care homes for older adults.
4	Communication Inequalities, Digital Health, Elderly Population, Health Technology, Mobile Phone, Smart Technology, Technology, Well-Being	Digital Health and Well-being	Articles that explore the intersection of communication inequalities, access to technology, and age-related barriers.
5	Alexa, Low-Income Senior Housing, Older Adults, Quality of Life, Smart Speaker Adoption, Technology Acceptance, Virtual Assistant, Voice Assistant	Digital Health Agents and Quality of Life	Articles on technology adoption and older adults’ quality of life.
6	Accessibility, Environmental Gerontology, Gerontechnology, Health-Well-Being Semi-Structured Interview, User Experience Design, Voice-Activated Persona	Health Technology and Usability	Articles designing and evaluating technology to enhance accessibility, health, and well-being among older adults.
7	Anthropomorphism, Conversational Interface, Low Technology Use, Ontology, Personification, Smart Speakers, Voice Assistants	Humanized Technology and Representation	Articles that humanized voice interaction in smart technology.
8	Communication, Intellectual Disability, Intelligent Personal Assistant, Speech Intelligibility, Cognitive Decline, Cognitive Training, Behavioral Health, Behavioral Intervention, Conversational Agent, Intervention, Intervention Research, Physical Activities, Physical Activity, Smart Devices	Health Behavior and Cognition	Articles that explores the connection between cognition, speech, and behavior

## Data Availability

No new data were created or analyzed in this study. Data sharing does not apply to this article.

## Abbreviations

The following abbreviations are used in this manuscript:

PRISMA	Preferred Reporting Items for Systematic Reviews and Meta-Analysis
OSF	Open Science Framework
MMT	Mixed-Methods Assessment Tool
QC	Quality Check
R	Relevance
